# Miscellany

**Published:** 1901-04

**Authors:** 


### Miscellany.

A Banded Logan Crown.—In this method of banding the Logan crown, the root is prepared as for a Richmond crown. The root-canal is enlarged and shaped to receive the pin of a Logan crown properly, using such a pin to determine the shape and fit. Make the band and cap and solder together as usual. Punch an oblong hole in the cap so as to receive the pin of the Logan crown. Place the cap and band in position on the root and insert an orange-wood peg in the canal similar in size and shape to the Logan crown pin, but fitting loosely so as to come away with the impression. Take an impression and bite all in one in plaster of Paris. Run the models and articulate in plaster. Withdraw the wooden peg. Select a Logan crown to suit the case, and with a carborundum or gem stone grind dry to articulate and also to form a close joint with the cap. Carbon paper can be used to determine high places in grinding. Then take a piece of thin platinum-foil and make cuts in it the shape of the capital letter I to receive the pin of the crown. Pass this over the pin and burnish down over the cervical end of the crown. This should lap over on the sides of the crown about one-sixteenth of an inch. Fasten the crown on the cap with hard wax or Parr's fluxed wax. Cut away the plaster and remove from the model. Invest crown downward and place a few pieces of solder around the pin and solder. This makes a crown with perfectly closed joints, and for naturalness, strength, and appearance is superior to a Richmond.

The crown may be cemented to the cap and then removed from the model, finished, and set without soldering.—McClain.

---

Gold Blindness, or Retinal Asthenopia, and its Treatment.—In gold blindness, the power to distinguish the gold from the walls of the tooth is lost after working a short time. This is caused by an overstimulation of the retina by the yellow rays of the gold. Exhaustion follows, and a scotoma, or blind spot, results, just as the refulgent rays of the sun will cause blindness by excessive stimulation. Looking at fused gold for a short time will produce similar results. All this work should be done only under the protection of properly tinted glasses.—Dr. L. Webster Fox, *Dental Cosmos.*

[After reading the excellent paper by Dr. Fox, the writer recalled the fact that he suffered from gold blindness while a student. All the above phenomena were experienced. The discovery was made that it was caused by getting the face too close to the work. This overtaxes the muscles, which results in the loss of the power of accommodation. A careful avoidance of close vision brought about a complete cure without glasses. Upon investigating this trouble among the students in the clinic in the University of Pennsylvania it is found to be quite common, and the same precaution which corrected the affliction in my own case corrects it in them, so far without exception.]

---

Inlays.—M. Chaupin demonstrated "inlays" in a slightly different method from any other at the International Dental Congress of 1900. The cavity was on the mesial surface of an upper right lateral. The teeth had been well separated. A piece of platinum-foil, large enough to cover the labial and palatine surfaces, and extending some distance below the incisive edge of the tooth, was pressed into the cavity in the usual way, and then folded over the lingual and labial surfaces and pinched together over the cutting edge of the tooth.

The larger piece of foil gives a great amount of stability to the whole matrix and there is not the same liability to disturb the matrix. The surface of the foil was painted over with a thin mixture of whiting and water up to the edges of the impression of the cavity. The reason for this is obvious and a good one. The inlay was fused over a gas blow-pipe, and the result was very good.—Dr. T. Mansell, *Dental Record.*

---

Formaldehyde Gas for disinfecting Books in Libraries.—For some time the belief has been current, doubtless not without good foundation, that diseases have been contracted by handling books in public libraries, which have become infected with the germs of some disease.

The New Jersey authorities have been the first to take preventive measures, because a case of scarlet fever had been shown to be transmitted by the agency of a book in a travelling library. Professor Mitchell, of the State Board of Health, has been conducting a number of experiments with formaldehyde gas on kindergarten toys and public school-books. So far it has been found the surest destroyer of microbes. It works quickly and effectively. This much has been demonstrated to the satisfaction of the experimenters, but the experiments are continued to discover the least amount of the gas that can be safely used, not only to kill the germs, but to make the books germ-proof, at least for a time.—*Medical Record.*

---

To finish Aluminum Plates.—At a clinic given at the recent meeting of the State Association at Louisiana, Mo., Dr. W. W. Flora, of Carthage, Mo., gave a demonstration on the use of caustic soda on aluminum. He uses a twenty-five per cent. solution on the cases after vulcanizing and finishing them. This gives a most beautiful finish and one that is pleasant to the sense of touch.—*Western Dental Journal.*

